# Image De-Identification Methods for Clinical Research in the XDS Environment

**DOI:** 10.1007/s10916-016-0431-7

**Published:** 2016-01-26

**Authors:** K. Y. E. Aryanto, G. van Kernebeek, B. Berendsen, M. Oudkerk, P. M. A. van Ooijen

**Affiliations:** Department of Radiology, Center for Medical Imaging - North East Netherlands (CMI-NEN), University of Groningen, University Medical Center Groningen, Groningen, The Netherlands; Department of ICT, University Medical Center Groningen, Groningen, The Netherlands; Rogan Delft, Veenendaal, The Netherlands

**Keywords:** XDS, XDS-I, Cross-enterprise document sharing, De-identification, Clinical Research, Patient data privacy

## Abstract

To investigate possible de-identification methodologies within the Cross-Enterprise Document Sharing for imaging (XDS-I) environment in order to provide strengthened support for image data exchange as part of clinical research projects. De-identification, using anonymization or pseudonymization, is the most common method to perform information removal within DICOM data. However, it is not a standard part of the XDS-I profiles. Different methodologies were observed to define how and where de-identification should take place within an XDS environment used for scientific research. De-identification service can be placed in three locations within the XDS-I framework: 1) within the Document Source, 2) between the Document Source and Document Consumer, and 3) within the Document Consumer. First method has a potential advantage with respect to the exposure of the images to outside systems but has drawbacks with respect to additional hardware and configuration requirements. Second and third method have big concern in exposing original documents with all identifiable data being intact after leaving the Document Source. De-identification within the Document Source has more advantages compared to the other methods. On the contrary, it is less recommended to perform de-identification within the Document Consumer since it has the highest risk of the exposure of patients identity due to the fact that images are exposed without de-identification during the transfers.

## Introduction

Medical imaging informatics has brought up numerous advantages and offers social, economic, clinical, and technical benefits for patient care. It plays an important role as it enables the establishment of an affordable yet high quality level of healthcare. Furthermore, the presence of imaging informatics in clinical trials and research promotes standardization in order to utilize the image for quantification and diagnosis and later enables the exchange or sharing of the acquired and derived data among enterprises appropriately in a quick and convenient manner.

Digital Imaging and Communication in Medicine (DICOM) [1] has been widely adopted nowadays as the universal standard of the medical image file format and communication protocol [2]. It is also claimed as a major step forward since it provides easy utilization of images in case of electronic storage and transfer in a multi-vendor environment [3]. DICOM introduces an explicit information object for various formats of (image) data and utilizes other standards to facilitate imaging integration in the health care enterprises [4].

To perform safe, secure and standardized clinical data sharing in a network of trusted partners, the Cross-Enterprise Document Sharing (XDS) profile [5] was initiated as one of the Integrating the Healthcare Enterprise (IHE) [6] profiles. However, solely adopting the protocol may lead to a security or interoperability flaw [7]. Some studies have provided modifications tailored to the protocol to enhance its sharing function [7, 8]. Later, a content profile to extend the XDS profile was developed to describe how the image and report data are shared between health enterprises. This extended profile is known as the Cross-Enterprises Document Sharing for Imaging (XDS-I) profile [9] and it has several advantages in reducing the network traffic and preventing image data duplication while still able to make the best use of medical imaging itself.

Most of the Trans-Institutional health information systems are still facing significant challenges of data transfer and communication [10]. Furthermore, when employed in a research setting, the implementation of XDS-I introduces the challenge of providing convenient and easy access to the shared study-related data from other enterprises without compromising patient privacy and patient data confidentiality. This involves the very important question of where to implement and execute the de-identification tasks within the XDS-I environment to provide an optimal solution for clinical research purposes both in sense of security and practicality. In this work, we investigate possible methodologies for image de-identification within the XDS-I environment, with their respective advantages and disadvantages, to provide improved support for image data exchange as part of clinical research projects.

## Materials and methods

As a widely used standard, DICOM provides the ability to communicate any kind of medical information together with its corresponding images from any type of (DICOM compatible) acquisition device. It standardizes the handling, storing, printing, and transmitting of information in medical imaging. It introduces explicit information objects for various formats of data [1] and utilizes other standards to facilitate imaging integration in the health care enterprise [4].

A DICOM file consists of two parts, the image itself (pixels data) and a header with meta-elements containing any information regarding the patient, institution, study, or pixel data. The header also involves public data such as patient name and number that will lead to the identity of a particular patient and thus introduces security and privacy issues since medical and administration staff have direct access to these data [11]. However, the risks are not only related to the header information directly connected to the identity of the patient. More general study information, also available in the header of a DICOM file, holds for example information about the study performed, the institutions that participate and the staff involved. The aggregation of this information from the DICOM header could on itself or combined with other sources of information also be used to track patient information and reveal the identity of a specific patient indirectly. This already introduces risks with respect to security and privacy within the walls of one institution. However, when images are shared among health enterprises without proper protection, the possible risks concerning data protection and securing patient privacy both increase.

De-identification of the DICOM tag elements should therefore be performed adequately by removing or changing all possibly sensitive information from the DICOM header. There are two known methods to perform such tasks, anonymization and pseudonymization.

Anonymization is claimed to be the most secure approach to ensure the privacy of DICOM data since it fully uncouples the data from the original patient [12]. It is used to completely remove confidential entries in the standard DICOM data dictionary, which could be used to derive the patient’s real identity, either by themselves or in combination with other entries. This method is aimed to gain an irreversible result in order to reduce the probability of revealing the patients identity.

Pseudonymization uses artificial identifiers to replace the most identifying fields within a data record. The purpose of adding these artificial identifiers or pseudonyms is to maker the data record less identifying One of the reasons to choose this method instead of anonymization is to provide an ability to trace back the real identity of the subject involved. This possibility of tracing back is useful when an appropriate follow up is necessary for the study or an aggregation of longitudinal data is important. Therefore, instead of removing data completely, a modification is done in such way that the associated parties (in most cases the principle investigator and/or data manager of a research project) are still able to obtain the real identity of the subject while attempts to identify the patient directly can be avoided. Thus, only necessary data are pseudonymized while the remaining fields should be made anonymous.

Integrating the Healthcare Enterprise (IHE) is an organization that develops and introduces profiles that are aimed at improving interoperability in healthcare. IHE profiles are not a technical standard. Therefore the initiated profiles are mainly an open infrastructure which is simple, easy, vendor independent, and free to implement by all enterprises involved [13]. The XDS-I profile was initiated by the IHE to provide a structure for an image sharing environment through a trusted network providing diagnostic related reports and information between healthcare enterprises. It uses existing standards in medical imaging, document management, and communication. It is a framework that describes the registration, query, retrieval, and publication of clinical documents.

**Fig. 1:**
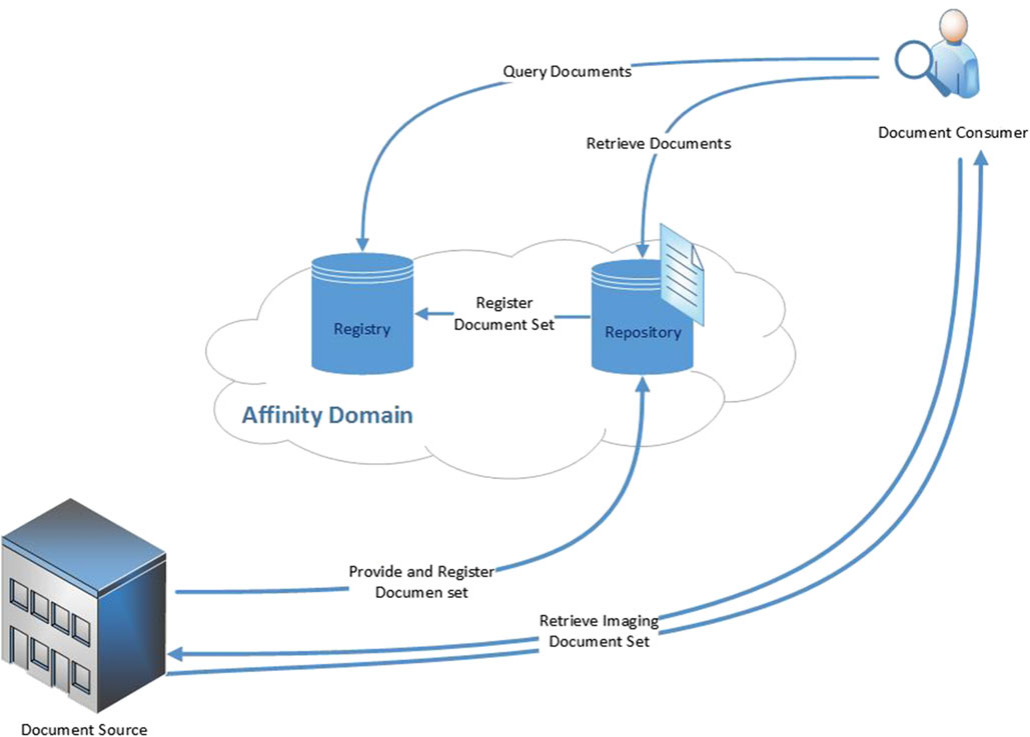
Actors and transactions in XDS-I. Document Consumer queries documents from the registrty and retrieve images either from repository or directly from source; Document Source provides the requested documents and transfers them to the consumer or repository; Document Registry indexes all published documents and repositories involved in data sharing; Document Repository handles the documents storage

XDS-I employs actors that can be grouped into Document Source, Document Repository, Document Registry, and Document Consumer. A Document Source is responsible for the document publishing and provides the clinical documents to a Document Consumer or the Document Repository. A Document Consumer is the actor that requests and retrieves documents from the XDS network, either from a Document Repository or a Document Source. The Document Repository handles the document storage in a transparent, secure, reliable, and persistent manner [14]. It is also responsible for delivering the requested documents to the Document Consumer, therefore the Document Repository should always be available. In XDS-I, images are not stored in the Repository. Instead, a small DICOM object called Key Object Selection (KOS) document containing a list of UID references is stored so that documents of interest can be easily found and retrieved from their original source location. The Document Registry is the actor that indexes all published documents and repositories involved in data sharing. Actors and transactions involved in the XDS-I environment are shown in Fig. 1.

The registry may contain a set of attributes of documents including sensitive data such as patient’s name, author’s institution, and author’s name. In the clinical research environment, the shared data are not allowed to have sensitive or private information embedded in it since that information can be used by other parties to trace the identity of the patient or study participant. Even though XDS-I offers a secure transfer over a trusted network, further efforts are needed to ensure that the sensitive information is eliminated or encoded. Furthermore, the original data must remain stored at the source domain and no duplication should be made in local or central repositories.

De-identification of DICOM data, as previously described, is the most common method to perform information removal. However, since de-identification is not a standard part of the XDS profiles, different methodologies could be used to enable the de-identification within XDS. Therefore, in this study different methodologies were defined and evaluated to decide how and where the de-identification should take place within an XDS environment used for scientific research.

## Results

**Fig. 2:**
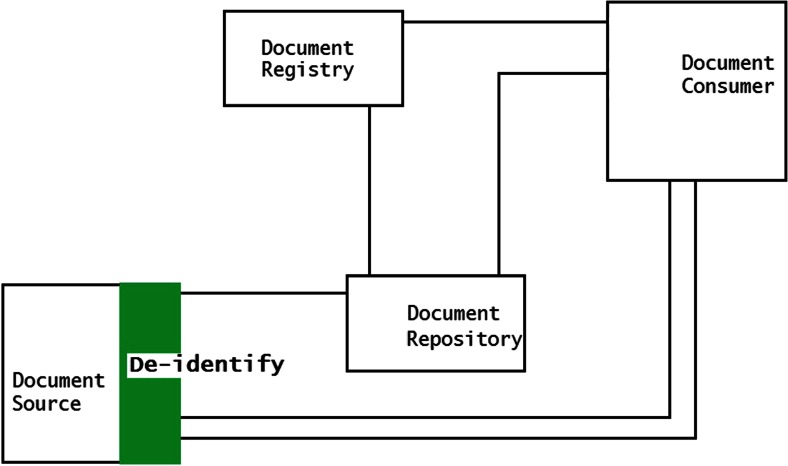
De-identification is performed within the Document Source (*shown as green area*), to ensure data are de-identified before leaving the source

**Fig. 3:**
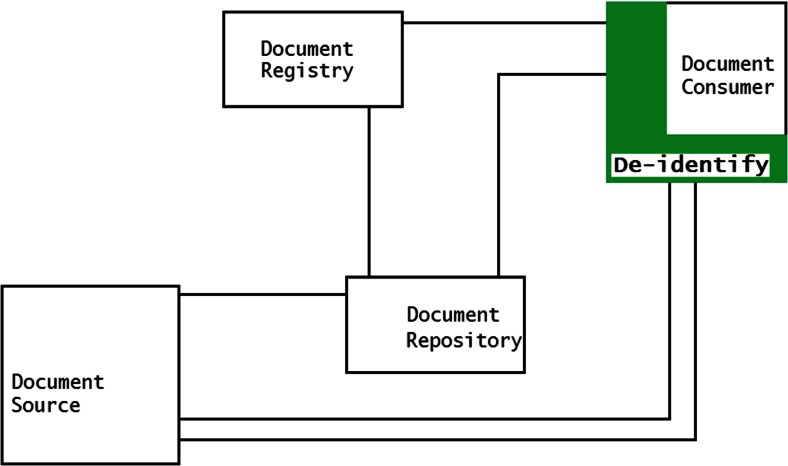
De-identification is performed in the Document Consumer (*shown as green area*). They are de-identified before being stored within the consumer’s domain

**Fig. 4:**
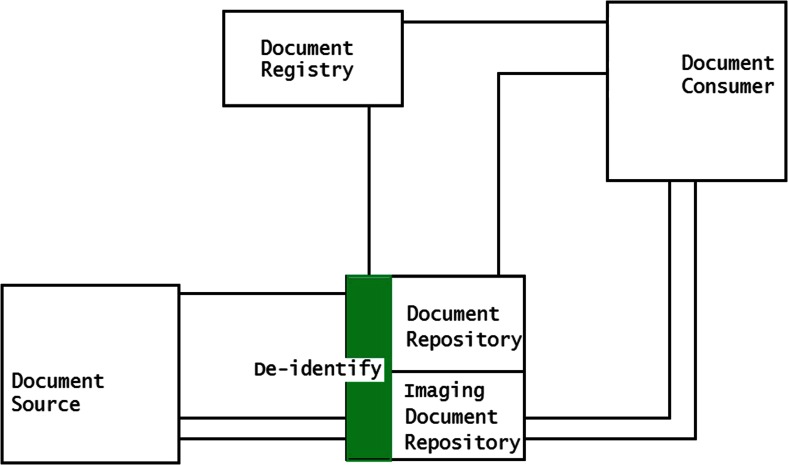
De-identification is performed within the Document Repository with additional required service placed between source and consumer (*shown as green area*), toensure data are de-identified before stored in the repository

Services can be implemented in three methods to perform the de-identification of the DICOM files within the XDS-I framework. First, de-identification can be performed within the Document Source before images are sent to Consumer (Fig. 2). Second, images are de-identified after images are received by the Document Consumer (Fig. 3). Third, de-identification is done between the Document Source and Document Consumer (Fig. 4). The last two methods require additional services to remove identifiable elements in the KOS documents and images itself to make sure that data are de-identified before being stored in the Repository.

Each of the methods has its own benefits and disadvantages. Several criteria were examined to see the effects of the position of de-identification to the whole process. Those criteria were the required additional processing time, the speed of processing, additional hardware, and the degree of identity protection during transfers.

De-identifying images within the Document Source requires more processing time within the source itself since the requested documents will be processed before they are sent to either Document Repository or Document Consumer. The waiting time of the Consumer becomes longer depending on the amount of images being processed in the de-identification queue.

**Fig. 5:**
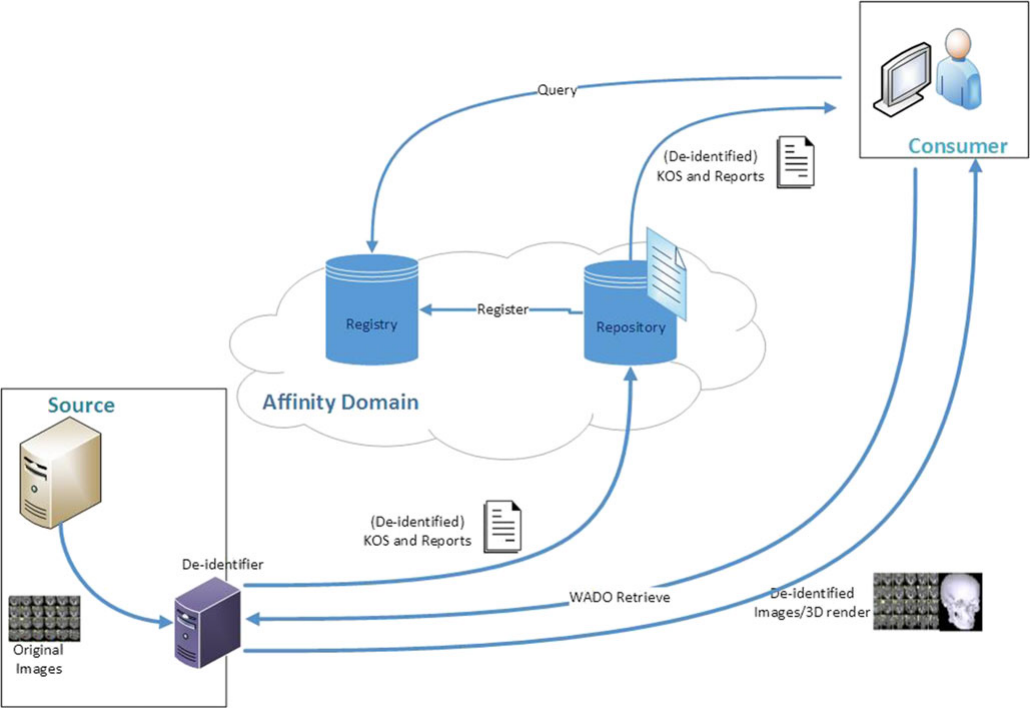
De-identification of data is done within the Document Source. De-identified documents are sent from the source

By processing images within the source, additional hardware and software are needed to be installed at each site of origin of the collected data. This implies that the amount of additional systems is at least equal to the amount of the enterprises which are involved in the data gathering of the research project. But this method has a potential advantage with respect to the exposure of the images to outside systems, because the images are ensured to be de-identified before leaving the site of origin. Figure 5 shows the document flows through this methodology.

**Fig. 6:**
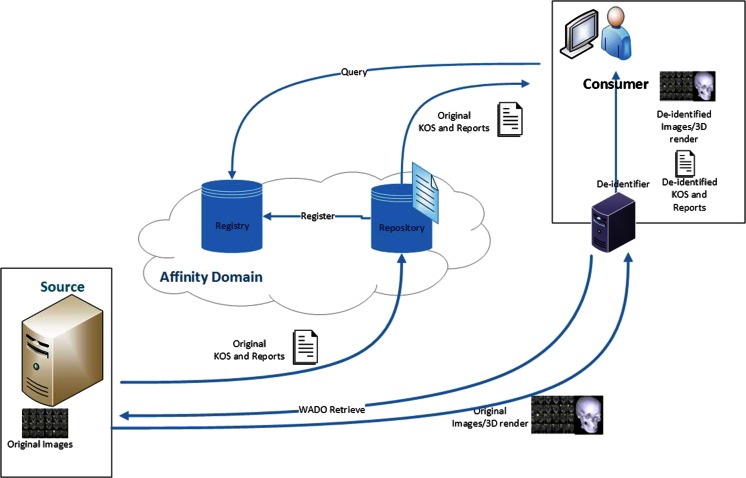
De-identification of data is done within the Document Consumer. Fully exposed original documents are sent through the network without prior de-identification

The second method is to perform de-identification within the Consumer. Document transfers require similar time compared to the normal flow of transfer within the XDS-I environment since no further actions will be taken before the data reach the Consumer. Additional time will only be needed in the Consumer itself since data are processed further within the Consumer as shown in Fig. 6. This method has drawbacks with respect to additional hardware and configuration requirements.

All de-identification processes will be done by the Consumer. Therefore, in case of multiple consumers, all consumers should be synchronized and use the same de-identification protocols which is not a practical solution. The biggest concern regarding this method is the fully exposed original documents with all identifiable data being intact before they reach the consumer, posing a higher risk of identity breach.

**Fig. 7:**
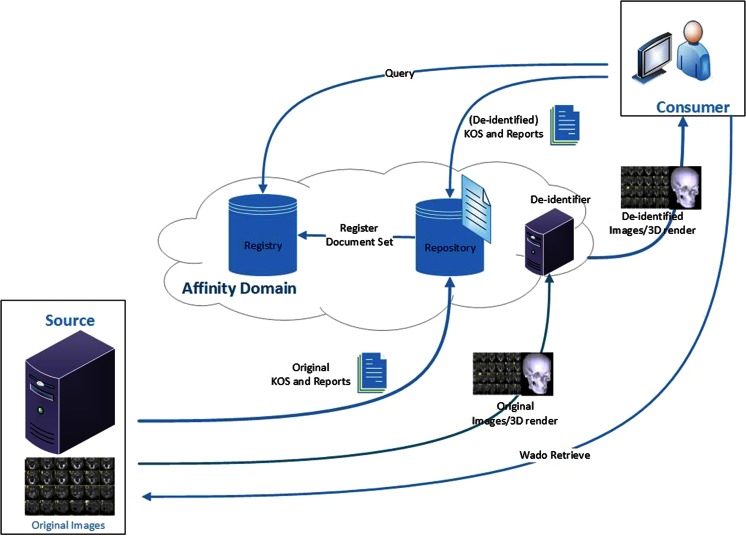
De-identification of data is done within the Affinity Domain. Images sent from the Document source are not de-identified

In the third method images are de-identified after being transmitted by the Document Source on their way to the Consumer. In this case, an additional service will be required as mediator between the Source and Consumer, for example a de-identification server within the Affinity Domain. This service will be used to receive images that are sent by the Document Source and appropriately process the documents before forwarding them to the Document Consumer. The mediators will take care of the entire de-identification process. Thus, the processing of images may have a hiccup within the affinity domain when there are many transactions in the queue due to many and/or large requests from the consumer or intensive transfers from the source. This method is shown in Fig. 7.

This method also has the risk of original images being exposed since they are transferred without any prior de-identification process from the site of origin into the affinity domain before reaching the de-identifier. It means that, upon request, the images from a publisher should always be sent to the repository and thus a consumer could not get the images directly from the source. Therefore, this method will change the scheme of XDS-I itself where images should only be exchanged directly by Source and Consumer without being kept by services in between. This would give a disadvantage in terms of simplicity of the transfer of the images and may hamper the workflow of the XDS-I domain.

Table **1** shows a summary of the three methodologies based on four criteria. It is shown that the second method has the highest risk of the exposure of patients’ identity due to the fact that images are exposed without de-identification during the transfers. Therefore, it is less recommended to do the de-identification at the consumer site.

**Table 1:** Summary of property ratings of all possible de-identification methodologies within the XDS-I environment

	De-identification within XDS Source	De-identification within XDS Consumer	De-identification within Affinity Domain
The speed of processing	+	+	+−
Processing time	+−	+	+−
Additional hardware required	−	−	+
Protection of identifiable elements during transfers	++	−−	−

−−very poor

−poor

+−fair

+good

++very good

## Discussion

### Processing time

Transfer of image data with the de-identification process done within the publisher domain may suffer from long waiting queues due to a large number of requests from multiple consumers at the same time, all requiring de-identification and transfer. However, in most research projects this scenario would not occur frequently and the retrieval of data in research studies are mostly not a time critical process. Comparing this to the method in which de-identification is done outside the Source, which likely is within the affinity domain, images will always go through the repository since the images cannot be sent directly from Source to Consumer.

### Workload and processing speed

Using the de-identification within the Consumer method, workload will depend on the requests a Source received. Therefore, the transfers of data are quite equally divided if all Sources receive requests with a nearly equal amount of data. A similar situation holds for the de-identification in the Consumer where Consumers request the same amount of data as the first option. Meanwhile, as previously mentioned, the transfer with de-identification being done within the Source and Consumer will be highly utilizing the de-identifier within the affinity domain causing high workload at that de-identifier, possibly leading to reduced processing speed.

### Additional hardware

Consideration of additional hardware and software will favor the de-identification between the Source and Consumer since there is a possibility to add only one additional de-identification system. The use of paid software will enhance the benefit of this option although open source alternatives do exist that could be used [15]. In case of hardware requirements, the method with de-identification being done within Source or Consumer will need as much additional services as the number of Sources or Consumers. Meanwhile method with de-identification being done in between requires a much higher specification hardware regarding processing power, memory and temporary storage to handle the centralized de-identification which will most likely lead to higher cost. However, the cost is not only dictated by the hardware but also by the cost of service and maintenance required for the hard- and software. The centralized node simplifies the service and maintenance of the servers lowering this part of the expenses.

### Identity protection

The chance of data being captured and directly reveal patient identity is higher when de-identification is performed after the images are transferred out of the source. The images are merely transferred without a prior de-identification process. Therefore, all sensitive information is still embedded in the images.

The method in which data are de-identified before being saved in the Document Consumer is regarded as less favorable since it has the highest risk of images being sent without any de-identification first.

An additional problem in the de-identification of medical image data is the burnt-in information sometimes included in the DICOM image during data acquisition or secondary capture. That kind of information cannot be removed easily using the DICOM header de-identification. Providing data with burnt-in patient information within the image itself is as harmful as transferring DICOM data with all original header elements left intact. Blacking out the pixels that contain sensitive information should be performed. In the method, this issue already exists since the data are transferred out from the Source without de-identification. Blacking out pixels will be performed either within Consumer with the second method or within affinity domain using the third option. Meanwhile, the issue still can be tackled when first method is used since the de-identification is done before images leave the XDS Source. However, this will lead to the caution of selecting the de-identification tools because not all toolkit can perform the task.

Furthermore, a three dimensional image reconstruction, mostly from CT or MR modalities of the head, combined with facial recognition software may also reveal the identity of patients [16, 17]. Several other methods are being investigated to be developed and provide a better protection to the patient data [18]

In order to keep the de-identification information well secured, a mapping database pointing to the origin of the images should be stored in such a way or place that only authorized parties are able to determine the original data. This kind of database can be placed at different locations. For example, it can be stored locally in the Source site, within the affinity domain, or be implemented through a trusted third party. When de-identification is done within the Source, it will be more practical if the records of the original metadata are kept within the Source itself and only deliver the new keys and a link to owner institution to the registry or the trusted third parties. Meanwhile, when de-identifications are done between Source and Consumer, the mapped identities can be sent back and recorded in Source or put it right away into database server within affinity domain.

## Conclusion

De-identification of images within the XDS-I environment is required when data sharing is implemented in clinical research. Comparison regarding the location of de-identification process to be placed within the XDS-I environment for clinical research has been done. Identity removal can most safely be done within the Document Source to prevent a privacy breach.
